# Eco-Friendly Production of Polyvinyl Alcohol/Carboxymethyl Cellulose Wound Healing Dressing Containing Sericin

**DOI:** 10.3390/gels10060412

**Published:** 2024-06-20

**Authors:** Massimo Mariello, Enrico Binetti, Maria Teresa Todaro, Antonio Qualtieri, Virgilio Brunetti, Pietro Siciliano, Massimo De Vittorio, Laura Blasi

**Affiliations:** 1 Center for Biomolecular Nanotechnologies, Italian Institute of Technology, 73100 Lecce, Italy; massimo.mariello@eng.ox.ac.uk (M.M.);; 2 Dipartimento Ingegneria dell’Innovazione, Università del Salento, via Monteroni, 73100 Lecce, Italy; 3 Institute for Microelectronics and Microsystems IMM-CNR, UOS di Lecce Via Monteroni c/o Campus Universitario Ecotekne-Palazzina A3, 73100 Lecce, Italy; 4 Institute of Nanotechnology NANOTEC-CNR, c/o Campus Ecotekne, Via Monteroni, 73100 Lecce, Italy

**Keywords:** hydrogel, wound healing, dressing, eco-friendly

## Abstract

Wound dressing production represents an important segment in the biomedical healthcare field, but finding a simple and eco-friendly method that combines a natural compound and a biocompatible dressing production for biomedical application is still a challenge. Therefore, the aim of this study is to develop wound healing dressings that are environmentally friendly, low cost, and easily produced, using natural agents and a physical crosslinking technique. Hydrogel wound healing dressings were prepared from polyvinyl alcohol/carboxymethyl cellulose and sericin using the freeze–thawing method as a crosslinking method. The morphological characterization was carried out by scanning electron microscopy (SEM), whereas the mechanical analysis was carried out by dynamic mechanical analysis (DMA) to test the tensile strength and compression properties. Then, the healing property of the wound dressing material was tested by in vitro and ex vivo tests. The results show a three-dimensional microporous structure with no cytotoxicity, excellent stretchability with compressive properties similar to those of human skin, and excellent healing properties. The proposed hydrogel dressing was tested in vitro with HaCaT keratinocytes and ex vivo with epidermal tissues, demonstrating an effective advantage on wound healing acceleration. Accordingly, this study was successful in developing wound healing dressings using natural agents and a simple and green crosslinking method.

## 1. Introduction

The skin is an important organ that plays an important role in protecting the body from the external environment; therefore, its protection can be decreased as a result of injury or damage. For this reason, many efforts have been devoted to the development of wound healing dressings to accelerate wound closure [1]. Ideally, materials for wound dressing should protect wounds from infection and provide an appropriate wound environment: e.g., keep the wound moist or provide mechanical protection, be easy to remove, and be biocompatible, biodegradable, and non-toxic [2].

Wound healing takes place efficiently in a wet environment, as fluid losses from wounded skin are almost 20 times greater than from normal skin [3]. Therefore, hydrogels are very effective as dressing materials because they can control the fluids lost since they hold large amounts of water, prevent scar formation, allow for cell proliferation and the epithelialization process, and possess a tissue-like structure and compatibility [4].

Furthermore, since the number of chronic wounds has risen due to the increase in the number of older adults [5], the production of wound dressings also continues to increase, resulting in high levels of pollution and waste. Therefore, finding clean, safe, and cost-effective techniques while maintaining healing properties remains a challenge in the development of wound dressings.

Green technology, which combines environmentally friendly processes and natural active ingredients, is a highly competitive field in pharmaceutical research and production development [6].

Freeze–thawing is a physical crosslinking technique used in the preparation of biomaterials. Avoiding toxic organic solvents and cross-linking agents, it has the advantage of yielding biocompatible and environmentally friendly materials [7,8].

Natural and synthetic polymers are often used as wound dressing materials [9]. Among them, poly(vinyl alcohol) (PVA), a water-soluble long-chain polymer obtained by the alcoholysis, hydrolysis, or ammonolysis of poly vinyl acetate, is a biocompatible and partially biodegradable material; it has high water absorption and good mechanical properties, e.g., a high elastic modulus and high mechanical strength [10,11].

Physical or chemical methods can be used to crosslink PVA. Chemical methods use crosslinking agents that react with the hydroxyl groups of PVA to form networks, which can affect the biocompatibility of the samples or interact with drugs or other filler compounds [12,13,14]. Indeed, physical methods of crosslinking PVA are preferred, and among them, freeze–thawing is the better choice since heat treatment or irradiation can affect drugs or biologically active compounds loaded in the wound dressing [15,16].

Carboxymethyl cellulose (CMC) is a water-soluble cellulose derivative, and due to its properties, including tunable biodegradability, potential antioxidant activity, biodegradability, and biocompatibility, it represents a highly versatile biomaterial [17].

Because of their minimal interference with new tissue formation and their high liquid absorption capacity, CMC can be used in various forms (hydrogels, scaffolds, etc.) and combinations (e.g., blended with other polymers) in biomedical engineering research, including wound healing and wound dressing applications [18,19].

Furthermore, its hydrophilic nature (due to its hydroxyl and carboxyl groups), allows it to be blended and cross-linked with other materials, such as synthetic polymers, natural polymers, and inorganic materials, thus enabling the preparation of innovative wound dressing biomaterials.

Accordingly, both PVA and CMC are suitable for use as dressing materials [20].

Furthermore, depending on the network’s composition and water vapor transmission (WVT), PVA/CMC blends have been demonstrated to be highly absorbent, with gel fraction values close to normal (healthy) skin tissue and commercial wound dressings, an important feature related to a balanced moisture microenvironment at the wound site [21]. In this context, the addition of a wound healing accelerating agent may improve the performance of wound dressings. Many natural active compounds have been used to produce wound dressings that ensure maximum recovery [22]. Among them, sericin is a natural protein synthesized by Bombyx mori, which is a common waste product from processing industries. Several hydrogels have been developed combining sericin with other polymers [23,24,25], since it is a biocompatible and biodegradable material with several biomedical properties, including antioxidant, anti-bacterial, anti-inflammatory, and wound healing acceleration effects [26,27,28,29]. It has been proven that sericin does not cause immunological reactions, has antioxidant properties, and can activate fibroblasts to promote collagen type I synthesis, leading to wound healing promotion [30].

In this study, we developed a wound healing dressing based on polyvinyl alcohol, carboxymethyl cellulose, and sericin using freeze–thawing as a crosslinking method. The physical, mechanical, and biological properties of the dressing were evaluated based on their structural analysis, elongation, Young’s modulus, cytotoxicity, and cell migration.

## 2. Results and Discussion

### 2.1. Eco-Friendly Preparation Method

**Figure 1:**
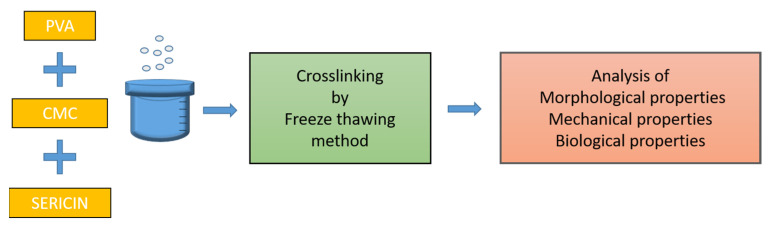
Illustration of the preparation process of PVA/CMC/SER wound healing dressings.

The proposed method for preparing the hydrogel wound dressings is distinguished by its environmental friendliness, ease of operation, low cost, and optimal throughput. The method is based on the sequential mixing of PVA with CMC, sericin, or both, in distilled water to prepare different formulations. After casting the prepared solutions into proper molds to shape the wound dressings, the crosslinking reaction was allowed to occur via freeze–thawing, i.e., each formulation was frozen at −80 °C for 16 h and then thawed at 25 °C for 8 h (Figure 1). After this step, the dressings were frozen at −80 °C until used. The method and the wound dressings’ compositions are described in more detail in the Section 4. Their mechanical, morphological, and biological properties were assessed through different techniques, which are described in the next sections.

### 2.2. Mechanical Properties of Wound Healing Dressings

The mechanical properties of hydrogel wound dressings, such as their strength, tensile strength, softness, flexibility, and elasticity, are essential for their applications since they should have good strength and elasticity to mimic the elasticity of skin during repair. The compression Young’s modulus was determined as the slope of the curves in the linear regime (low strains), and it was used for evaluating and comparing the mechanical properties of the as-prepared hydrogel wound dressing materials.

**Figure 2:**
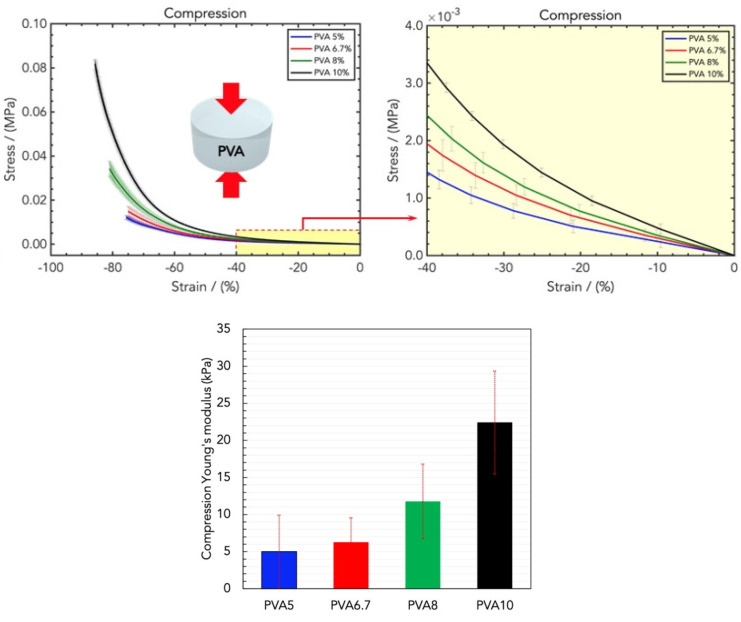
(**Top**) Stress/strain curves resulting from DMA (compression) analysis of wound dressings made of different concentrations of PVA in water, namely PVA5, PVA6.7, PVA8, and PVA10. (**Bottom**) Comparison of the compression Young’s moduli for the four PVA concentrations.

The rigidity of the hydrogel increased with the PVA loading due to the increases in the hindrance of the polymeric chains and the number of reticulation sites (Figure 2, top) [31]. In particular, the elastic moduli were (5.01 ± 4.00) kPa, (6.26 ± 3.32) kPa, (11.76 ± 5.03) kPa, and (22.41 ± 6.95) KPa for PVA5, PVA6.7, PVA8, and PVA10, respectively (Figure 2, bottom).

**Figure 3:**
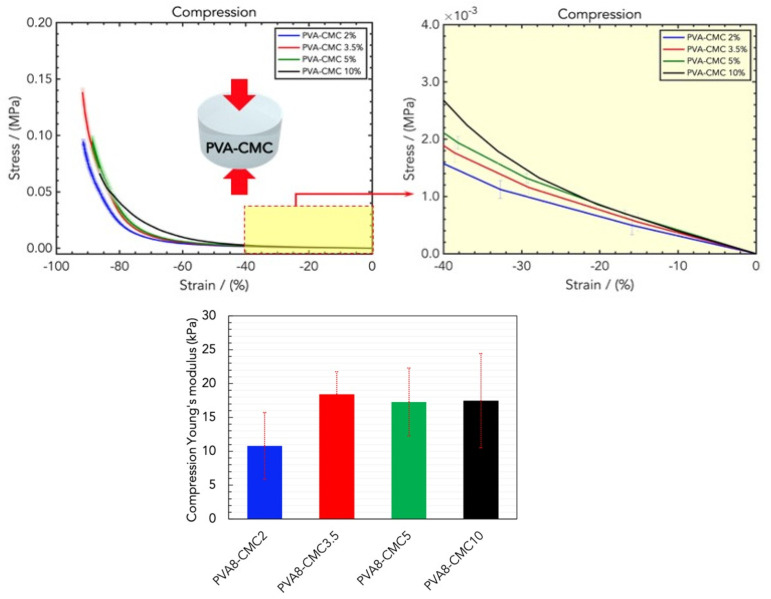
(**Top**) Stress/strain curves resulting from DMA (compression) analysis of wound dressings made of PVA8 in water and different concentrations of CMC, namely PVA8/CMC2, PVA8/CMC3.5, PVA8/CMC5, and PVA8/CMC10. (**Bottom**) Comparison of the compression Young’s moduli for the four CMC concentrations.

Fixing the amount of PVA8, CMC was added, and the linear elastic modulus was (10.81 ± 2.26) kPa, (18.42 ± 6.33) kPa, (17.38 ± 3.57) kPa, and (17.49 ± 10.37) kPa for PVA8/CMC2, PVA8/CMC3.5, PVA8/CMC5, and PVA8/CMC10 (Figure 3); therefore, the CMC loading increased the rigidity. The upper bound of this increase is represented by the rigidity of the PVA at the maximum tested concentration. In particular, the PVA8/CMC3.5 exhibited the best combination of linear elasticity and strain capability. With CMC loadings higher than that of PVA8/CMC3.5, the ultimate stress and strain exhibited by the prepared hydrogels decreased (Figure 3, bottom); therefore, we chose this combination. 

**Figure 4:**
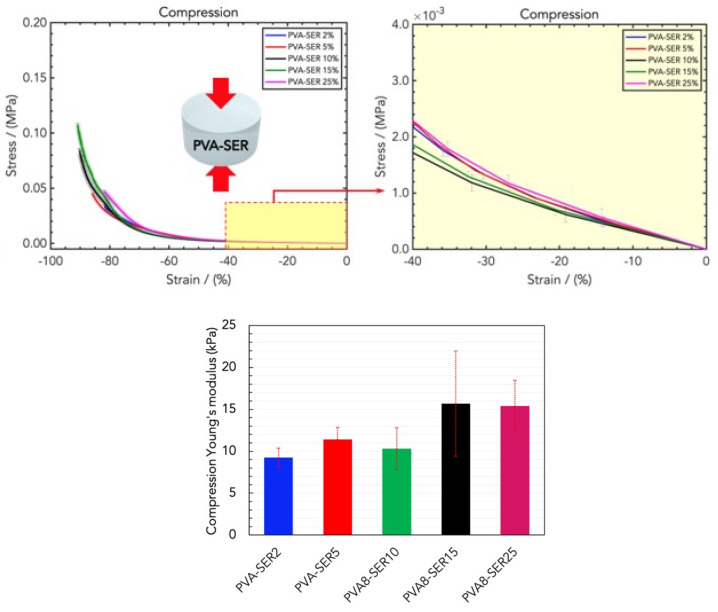
(**Top**) Stress/strain curves resulting from the DMA (compression) analysis of wound dressings made of PVA/water and different concentrations of sericin (SER), namely PVA8/SER2, PVA8/SER5, PVA8/SER10, PVA8/SER15, and PVA8/SER25. (**Bottom**) Comparison of the compression Young’s moduli for the five SER concentrations.

Before analyzing the three-component blend, in order to evaluate the effect of adding sericin on the rigidity of the hydrogel material, PVA8 samples were loaded with SER in order to obtain different combinations of PVA and sericin. (Figure 4, top). The addition of sericin induced an increase in the linear elastic rigidity, i.e., (9.23 ± 1.13) kPa, (11.43 ± 1.43) kPa, (10.31 ± 2.51) kPa, (15.67 ± 6.30) kPa, and (15.39 ± 3.08) kPa for PVA8/SER2, PVA8/SER5, PVA8/SER10, PVA8/SER15, and PVA8/SER25, respectively (Figure 4, bottom). In particular, for higher values of SER, the blend exhibited an improved elastic modulus, showing more compactness and also resulting in a more slippery effect on its surfaces. 

**Figure 5:**
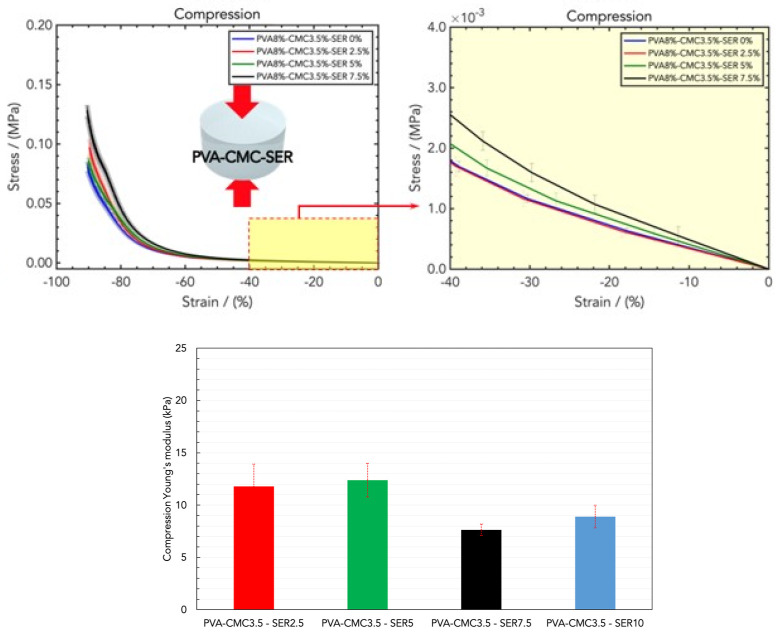
(**Top**) Stress/strain curves resulting from DMA (compression) analysis of wound dressings made of PVA8/CMC3.5 and different concentrations of sericin (SER), namely PVA8/CMC3.5/SER2.5, PVA8/CMC3.5/SER5, PVA8/CMC3.5/SER7.5, and PVA8/CMC3.5/SER10. (**Bottom**) Comparison of the compression Young’s modulus values for the four SER concentrations.

The combination of PVA with CMC and sericin provides optimal results in terms of the mechanical properties, as illustrated in (Figure 5). With fixed amounts of PVA and CMC (PVA8/CMC3.5), different SER loadings were tested, namely PVA8/CMC3.5/SER2.5, PVA8/CMC3.5/SER5, PVA8/CMC3.5/SER7.5, and PVA8/CMC3.5/SER10. The compression Young’s modulus values were as follows, respectively: (14.15 ± 2.13) kPa, (11.79 ± 1.61) kPa, (12.39 ± 0.55) kPa, (7.64 ± 1.09) kPa, and (8.90 ± 4.67) kPa (Figure 5, bottom). The effect of the addition of sericin to the PVA/CMC blend was a decrease in the linear elastic compressive rigidity, which is in contrast to the effect of sericin on PVA. This can be ascribed to sericin, with its lateral side groups in amino acids that facilitate blending with the polymeric chains of CMC, thus resulting in a softer and more elastic microstructure [32,33]. With a fixed amount of PVA (PVA8), the compression tests revealed the optimal loading of PVA8/CMC3.5 and that of sericin higher than in PVA8/CMC3.5/SER7.5. The compression results related to the hydrogel samples containing a higher concentration of sericin, namely PVA8/CMC3.5/SER15, were not reported since they were very slippery, which prevented a correct measurement. 

**Figure 6:**
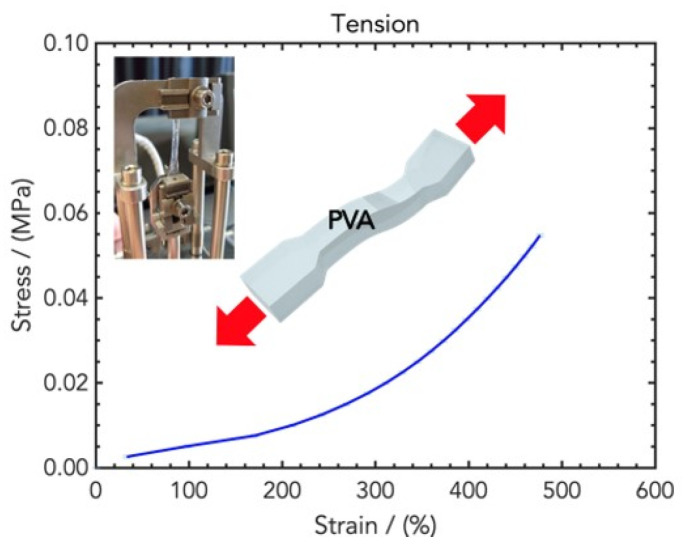
Stress/strain curve resulting from DMA (tension) analysis of wound dressings made of PVA/water.

Tensile tests were performed to compare the tensile rigidity of the hydrogels, since they need to be applied on the skin for wearable applications, and their stretchability determines their conformality and skin-adaptability. The resulting stress/strain curves are displayed in Figure 6: as expected, the curves present a higher data dispersion for high strains due to the high variability of the stretched elastomeric materials. The tensile Young’s moduli in the linear low-strain regime and in the high-strain regime were determined from the slope of the curves, whereas the ultimate stress and the strain at break provide an indication of the stretchability of the hydrogel patches.

**Figure 7:**
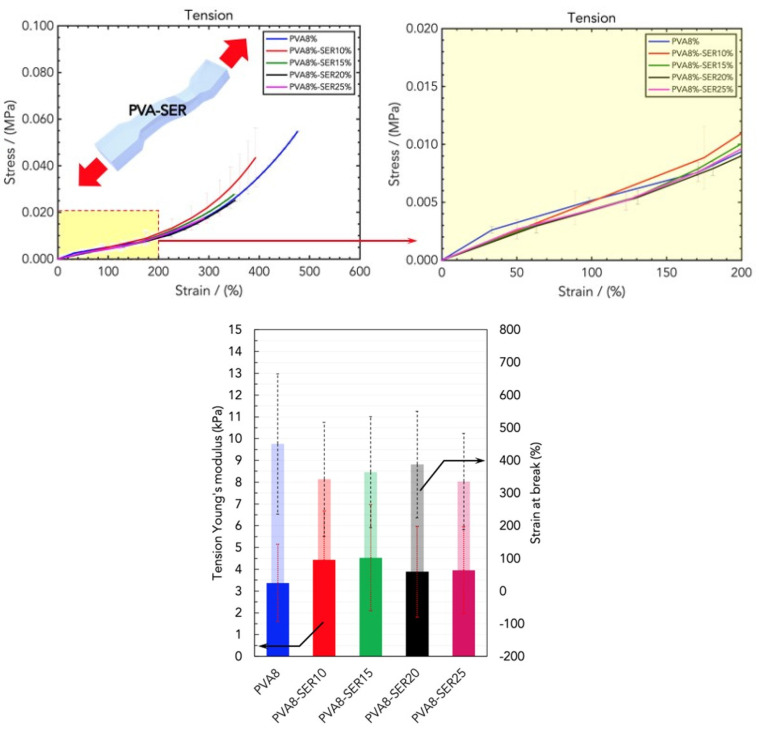
(**Top**) Stress/strain curves resulting from DMA (tension) analysis of wound dressings made of PVA and different concentrations of SER, namely PVA8/SER10, PVA8/SER15, PVA8/SER20, and PVA8/SER25. (**Bottom**) Comparison of tensile Young’s modulus and strain at break for the five SER concentrations.

The sericin loading in PVA8/SER10, PVA8/SER15, PVA8/SER20, and PVA8/SER25 improved the low-strain rigidity but negatively affected the other parameters (Figure 7), allegedly due to the mechanical robustness and low elasticity of the protein, which hardened the texture and compactness of the hydrogels. In addition, higher sericin loadings induced a slippery effect on the gripping, which produced a lower Young’s modulus value. 

**Figure 8:**
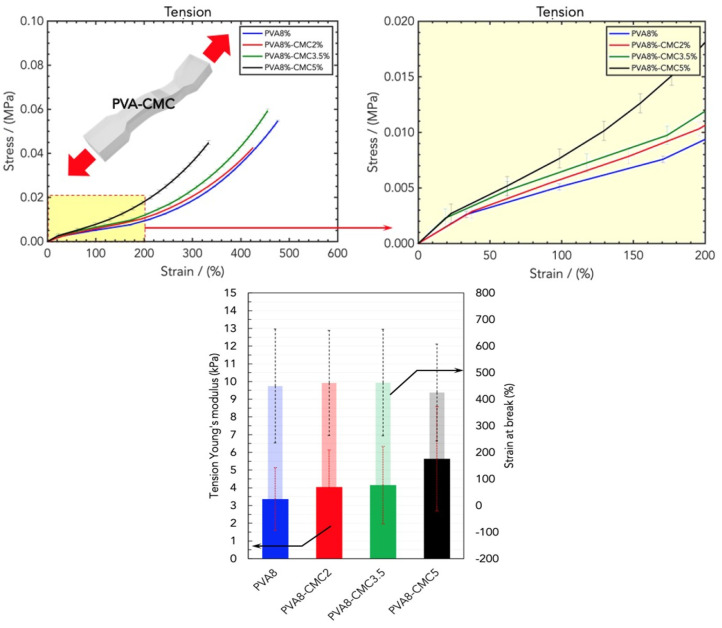
(**Top**) Stress/strain curves resulting from DMA (tension) analysis of wound dressings made of PVA8 and different concentrations of CMC, namely PVA8/CMC2, PVA8/CMC3.5, and PVA8/CMC5. (**Bottom**) Comparison of tensile Young’s modulus and strain at break for the four CMC concentrations.

On the other hand, the CMC in PVA8/CMC2, PVA8/CMC3.5, and PVA8/CMC5 did not significantly affect the tensile mechanical parameters (Figure 8). This is in contrast to the results of the compression analysis, according to which both the CMC and sericin improved the PVA’s mechanical resistance: this difference can be ascribed to the different properties (especially its low tensile elasticity) of the protein with respect to the other polymers.

**Figure 9:**
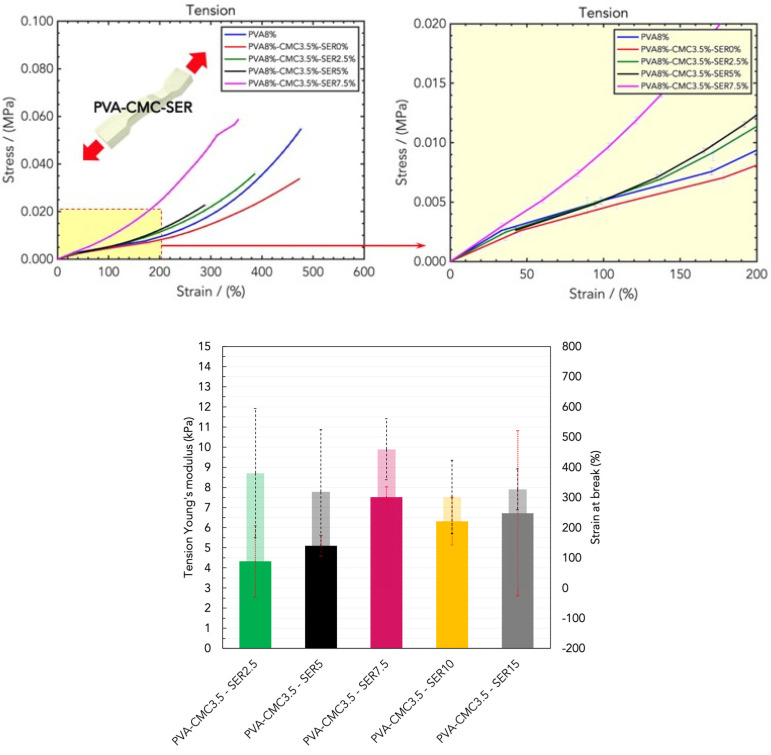
(**Top**) Stress/strain curves resulting from DMA (compression) analysis of wound dressings made of PVA8/CMC3.5 and different concentrations of sericin (SER), namely PVA8/CMC3.5/SER2.5, PVA8/CMC3.5/SER5, PVA8/CMC3.5/SER7.5, PVA8/CMC3.5/SER10, and PVA8/CMC3.5/SER15. (**Bottom**) Comparison of the compression Young’s moduli for the five SER concentrations.

To evaluate the optimal sericin loading in tension mode, tensile tests were performed on hydrogel dressings made of the PVA8/CMC3.5 blend with different amounts of sericin. The results are reported in Figure 9 and reveal a steady increase in the tensile Young’s modulus with an optimal amount of sericin higher than in the PVA8/CMC3.5/SER7.5 blend, since these samples exhibited linear elastic rigidity, but maintained a high stretchability strain to break. In particular, the PVA8/CMC3.5/SER10 and PVA8/CMC3.5/SER15 blends had Young’s moduli of (6.32 ± 3.18) kPa and (6.71 ± 3.41) kPa, and strains to break of (301 ± 139)% and (326 ± 155)%, respectively.

Therefore, taking into consideration the results from the tensile and compression tests, as well as considering sericin’s biological activity, we converged two formulations: PVA8/CMC3.5/SER10 and PVA8/CMC3.5/SER15, namely SER1 and SER2, which have excellent stretchability and compressive properties, similar to those of human skin, and high contents of sericin [34].

### 2.3. Morphological Characterization of the Wound Dressings

Scanning electron microscopy (SEM), used to characterize hydrogel microarchitectures [35], provided a detailed morphological analysis of the wound dressings, from the overall structure of the material to the cross-sections.

**Figure 10:**
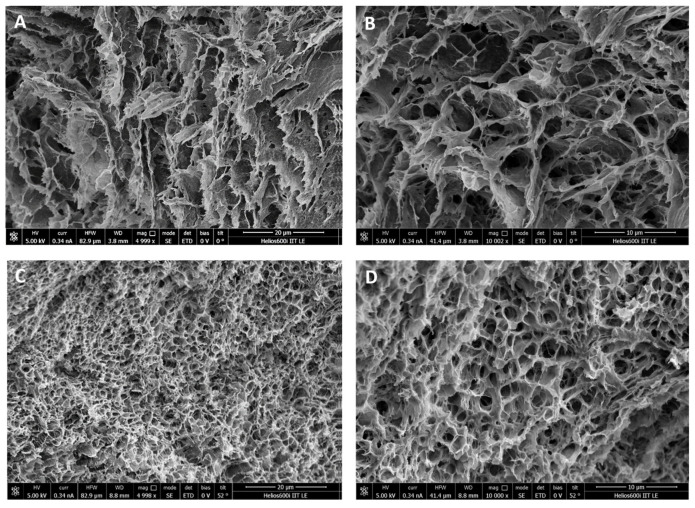
SEM micrographs of freeze-dried SER1 (**A**,**B**) and SER2 (**C**,**D**) at different magnifications.

Figure 10 shows SEM micrographs of the freeze-dried SER1 (Figure 10A,B) and SER2 (Figure 10C,D) hydrogel wound healing dressings at different magnifications. Both samples are characterized by a honeycomb-like macroporous structure, which is essential for biomedical application since it provides space for oxygen and nutrient transportation and cell growth [36]. Furthermore, the SER1 dressing had fewer pores than SER2, which exhibited a three-dimensional structure with different pore sizes. These results show that the average pore diameter decreased as the percentage of sericin increased. According to the literature, the highly porous structure of SER2 suggests its ability to absorb wound exudate and promote skin cell adhesion and spreading for the re-epithelialization of injured skin [37].

SER2 addressed two important issues, i.e., mechanical strength that mimics skin characteristics and suitable porosity to absorb wound exudate. Therefore, in the following sections of this manuscript, unless otherwise indicated, we always refer to SER2 (PVA8/CMC3.5/SER15) as the wound dressing material.

### 2.4. Biological Properties of Wound-Healing Dressings

The SER2 hydrogel wound dressing is made of three components, one synthetic polymer, PVA, a semi-natural CMC, and a protein, the silkworm sericin (SER). PVA was used because of its biocompatibility and good mechanical properties and because it can be crosslinked by physical methods. Carboxymethyl cellulose (CMC) has excellent water-absorbing and swelling capacities, is able to blend with other polymers, and is characterized by antioxidant activity [38].

SER is a water-soluble protein with mitogenic and cytoprotective effects on keratinocytes and fibroblasts, as well as on tissue repair and skin development [39,40]. Furthermore, amines, hydroxyl, and carboxyl groups enable the crosslinking of sericin with other biomaterials (e.g., PVA), thus improving mechanical performance.

In order to assess the biological properties of the developed SER2 as wound dressing material, in vitro and ex vivo experiments were performed.

**Figure 11:**
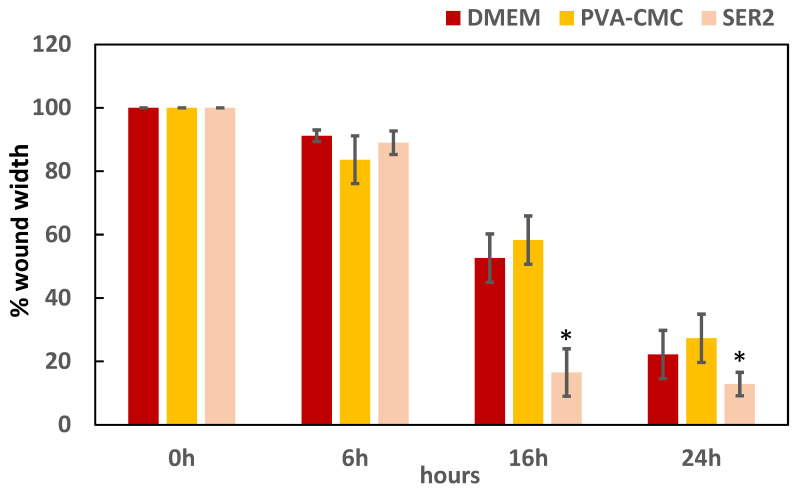
HaCaT cell scratch assay results after exposure to DMEM (control), PVA8/CMC3.5, or SER2 wound dressing material. The asterisks indicate a statistically significant difference (*p*< 0.05).

A scratch assay was used to investigate the effects of the developed wound dressing material on cellular proliferation and migration. Human keratinocyte (HaCaT) cells were used since they proliferate and migrate to close the wound and restore the epithelial layer [41]; a higher rate of cell migration promotes a faster wound healing process. The HaCaT cells were grown until 80% confluence, and scratch wounds were created and photographed over time up to 24 h (T0, T6, T16, and T24). The cell-free area was assessed, and the cell migration rate was calculated as the decrease in the cell-free area (Figure 11).

Quantification of the wounded area revealed that incubation with the SER2 dressing significantly enhanced the migration of HaCaT cells to the scratch area after 16 h (*p* = 0.001) and after 24 h (*p* = 0.0004), compared to the control (DMEM) and PVA8/CMC3.5 groups, whereas, no significant differences were found after 6 h when comparing the treated and CTRL groups (*p* = 0.24).

The skin is composed of two layers: the epidermis and the dermis, separated by the basement membrane made of collagen IV and laminin. The outer layer (epidermis) consists mostly of keratinocytes, melanocytes, Merkel cells, and Langerhans cells, and the dermis is composed of fibroblasts and is rich in extracellular matrix (ECM) components (e.g., collagen I–III, elastins, and proteoglycans) [42]. Therefore, cutaneous wound healing involves interactions between dermal fibroblasts and epidermal keratinocytes as well as cell–extracellular matrix interactions.

Fibroblasts are important in regulating the inflammatory phase, since the early stage of wound healing is characterized by crosstalk between fibroblasts and immune cells. Afterward, fibroblasts are responsible for producing extracellular matrix (ECM) components and determining crosstalk with endothelial cells and keratinocytes [43].

The current study describes the wound healing experiments conducted in EpiDerm Full Thickness (EFT) models, which are widely used in dermatological research, including wound healing, to reduce animal use in research. This in vitro human skin model consists of human epidermis comprising human keratinocytes and fibroblasts. Epidermal wounds, induced by means of a 2mm punch biopsy, were evaluated after incubation with PVA8/CMC3.5 and SER2 (PVA8/CMC3.5/SER15) as hydrogel wound dressing materials. The in situ re-epithelization was qualitatively analyzed by immunostaining with markers of dermal fibroblasts (vimentin) and keratinocytes (keratin 14). To distinguish the keratinocytes from the fibroblasts in the co-culture, the keratinocytes were stained green, and the fibroblasts were stained red.

**Figure 12:**
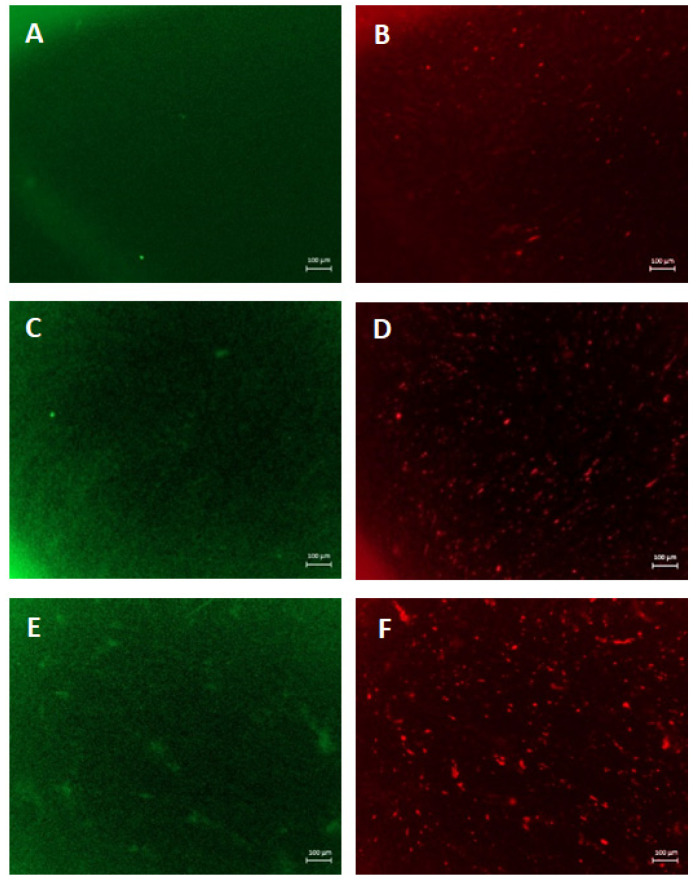
In situ staining of wounded EpiDermFT. Tissues were wounded with a 2 mm punch biopsy and immunostained with Keratin 14 (green fluorescence) (**A**,**C**,**E**) and vimentin (red fluorescence) (**B**,**D**,**F**). (**A**,**B**) Fluorescence micrographs of the control sample; (**C**,**D**) fluorescence micrographs tissues incubated with dressing containing only PVA and CMC; (**E**,**F**) wound incubated with SER2 dressing. Scale bars represent 100 μm.

**Figure 13:**
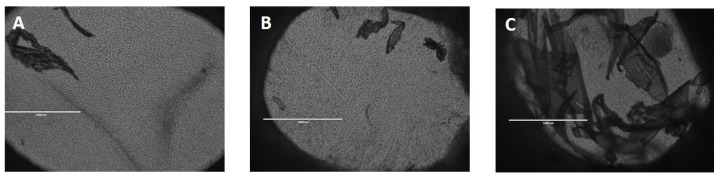
Optical images of small epidermal wounds induced in the model using a 2 mm punch biopsy. (**A**) Control sample; (**B**) wound incubated with PVA8/CMC3.5 dressing; (**C**) wound incubated with SER2 dressing. Scale bars represent 1000 μm.

**Figure 14:**
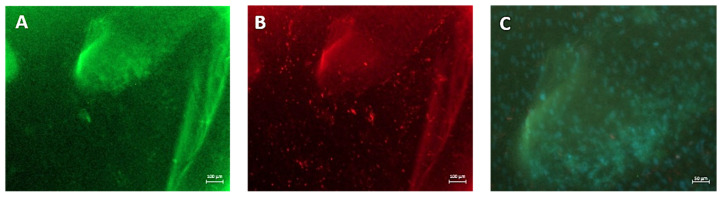
In situ staining of skin-like structures taken from epidermal wounds incubated with SER2 dressing and immunostained with (**A**) Keratin 14 (green fluorescence) and (**B**) vimentin (red fluorescence). (**C**) High-magnification details of the skin-like structures: blue shows DAPI-stained nuclei, and green shows Keratin 14 immunostaining. Scale bars represent 100 μm for (**A**,**B**), and 50 μm for the magnified image (**C**).

Figure 12 shows the wounded EpiDermFT immunostained with Keratin 14 (green fluorescence) (Figure 12A,C,E) and vimentin (Figure 12B,D,F). On day 7, the vimentin-positive fibroblasts overgrew in the samples incubated with SER2. They highly populated the induced wounds within the EFT tissues (Figure 12F), whereas few red spots were visible within the wound areas in the control samples and tissues incubated with PVA8/CMC3.5, as shown in Figure 12B,D respectively, thus demonstrating the increase in fibroblast migration across the wound, induced by the presence of sericin in the hydrogel wound dressing. Furthermore, the green fluorescent signal indicates that keratinocytes were present in the region of the wound (Figure 12A,C,E). Notably, as shown in Figure 13, in the sample treated with SER2, many skin-like structures covered the wound (Figure 13C). The green fluorescence shows that CK-14 positive keratinocytes were present in these structures; afterward, they were then counterstained with DAPI to highlight the cell nuclei (Figure 14). Therefore, in the EFT models with SER2, which contains sericin, cell migration and proliferation increased compared to the samples incubated with PVA8/CMC3.5 wound dressings and the controls (DMEM).

Our results agree with the results reported by Tariq et al. [44], who demonstrated the wound healing effects of sericin in diabetic mice; further studies demonstrated that sericin increased cell attachment and migration, the proliferation of keratinocytes and fibroblasts, and collagen synthesis, thus promoting wound healing in humans without the production of pro-inflammatory cytokines [45,46,47,48,49].

Although a PVA/CMC/SER hydrogel and its physicochemical characterization (gel fraction, water absorption, FT-IR, etc.) have been already proposed [23], here, we report for the first time the mechanical properties and biological activity of an optimized SER2 blend to be used for wound dressing application.

## 3. Conclusions

This study demonstrates the successful eco-friendly production of hydrogel wound dressings based on polyvinyl alcohol, carboxymethyl cellulose, and sericin. The preparation method is environmentally friendly and based on freeze–thawing as a crosslinking method. The hydrogel wound dressings exhibited elasticity, non-toxicity, and biocompatibility and possessed high potential for wound healing acceleration, as demonstrated by in vitro and ex vivo experiments. Further improvements of the proposed wound healing dressing can be envisioned by tuning the mechanical and controlled drug release properties to improve cell migration or to obtain effective timing patterns for wound healing. Finally, additional properties, such as oxygen permeability and skin adhesion, as well as in vivo efficacy and safety studies, will be investigated in future studies.

## 4. Materials and Methods

### 4.1. Materials

Poly (vinyl alcohol) (PVA, MW 146,000–186,000), sodium carboxymethyl cellulose, and sericin were purchased from Sigma Aldrich. HaCaT keratinocytes were purchased from Cell Line Service GmbH (Eppelheim, Germany) and cultured in 5% CO_2_ at 37 °C in regular Dulbecco’s Modified Eagle’s Medium (DMEM) (Corning) supplemented with 10% heat-inactivated fetal bovine serum (Corning), glutamine (2 mM), penicillin (100 U⁄ml) (Corning), and streptomycin (100 mg/ml) (Corning). Trypsin was purchased from Sigma Aldrich. EpiDermFT^TM^ tissues (EFT-412) were provided from the MatTek Corporation (Ashland, MA, USA); Dulbecco’s Modified Eagle (DMEM)-based medium for maintaining cultures was supplied by the manufacturer (EFT-400-MM). Anti-Cytokeratin 14 antibody, Recombinant Anti-Vimentin antibody, Goat Anti-Rabbit IgG H&L (Alexa Fluor^®^ 647) (ab150079), and Donkey Anti-Mouse IgG H&L Alexa Fluor^®^ 488) (ab150105) were procured from Abcam and used according to the manufacturer’s instructions.

### 4.2. Hydrogel Wound Healing Dressing Preparation

Hydrogel wound healing dressings with different compositions were prepared by mixing calculated amounts of PVA 10%wt, CMC 4%wt, and sericin 12%wt. The starting solutions of PVA and CMC were prepared by stirring the powders in water at 90 °C for at least 1 h. Such solutions were extremely viscous and, therefore, were handled at a high temperature, whereas the sericin was easily dissolved in water at room temperature. The compositions of the tested samples are reported in Table 1.

All mixtures were uniformly poured into a 12-well plate. The crosslinking reaction of the tested solutions was allowed to proceed by the freeze–thawing method. Each formulation was frozen at −80 °C for 16 h, and then thawed at 25 °C for 8 h. This process was counted as 1 cycle. Each formulation was prepared using 1 cycle of the freeze–thawing method and then frozen and stored long-term at −80 °C until it was used, retaining its mechanical properties and biological activity.

### 4.3. Mechanical Tensile and Compression Measurements

**Figure 15:**
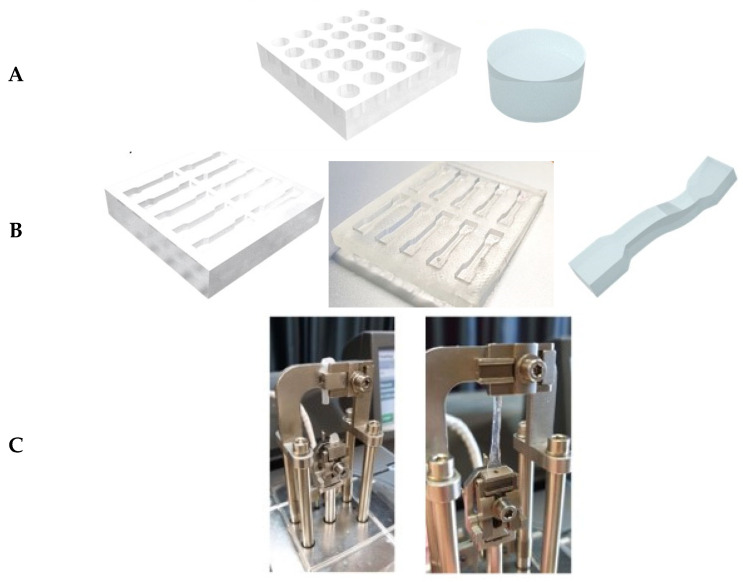
Samples used for the DMA analysis. (**A**) Circular samples for compression tests. (**B**) Dog-bone samples for tensile tests. (**C**) Real photos of the hydrogel dog-bone samples under tensile stretching.

Mechanical tensile and compression tests were conducted on the non-swollen hydrogels through dynamic mechanical analysis (DMA). The samples were removed from freezing conditions half an hour before the mechanical analyses. For the compression tests, the samples were shaped into circles with a 6.5 mm diameter and a 3.5 mm height (Figure 15A). The tests were performed with a ramp force of 1 N/min to an upper limit of 18 N and a pre-load of 0.01 N.

For the tensile tests, the samples were shaped according to the ASTM E8 standard for tensile test specimens [50]. For this purpose, a polylactic acid (PLA) 3D-printed master was used to create a PDMS mold with the right shapes (Figure 15B). In particular, the gauge length of the shaped hydrogels was 15 mm, whereas the ends were wider to favor the gripping of the clamps in the tensile machine (Figure 15C). The width and thickness of the specimens were 3 mm and 2.5 mm, respectively. In the case of too-slippery samples, an adhesive rough tape was used on the inner side of the clamps to improve the grip. The tests were performed with a ramp force of 0.5 N/min up to the failure of the samples (Figure 15C) and a pre-load of 0.01 N. All the results are an average of the five tested samples.

### 4.4. Morphological Characterization by SEM Analysis

Morphological analysis of the freeze-dried SER1 (PVA8/CMC3.5/SER10) and SER2 (PVA8/CMC3.5/SER15) wound dressings were performed by means of a scanning electron microscopy-focused ion dual beam (SEM-FIB) using the FEI Helios NanoLab 600i Dual Beam. All samples were sputter-coated with a thin gold film prior to SEM analysis.

### 4.5. Wound Scratch Assay

For the wound healing and cell migration assays, HaCaT cells were plated into a 12-well plate at a concentration of 1 × 10^5^ cells containing Dulbecco’s modified Eagle’s medium supplemented with 10% fetal bovine serum (FBS) and incubated overnight at 37 °C in a humidified 5% CO_2_ atmosphere. We left them until they reached 80% confluence. Afterward, the DMEM was completely removed, and the adherent cell layer was scratched with a sterile pipette tip; we also made horizontal reference lines to have a grid for alignment to obtain the same field for each image acquisition cycle. Cellular debris were removed by washing with PBS. Then, to the treated groups, the PVA8/CMC3.5 and SER2 (PVA8/CMC3.5/SER15) dressings were added. The control cells received only fresh medium. The cells were then incubated at 37 °C in a humidified 5% CO_2_ atmosphere. Then, the images of the scratch area were captured at 0 h (just after scratching cells), 6, 16, and 24 h using the AMG Evos FL Fluorescence microscope.

We determined the scratch area, wound coverage of the total area, and the average and standard deviation of the scratch width by means of ImageJ software (Version 1.54j) (https://imagej.net/ij/download.html accessed on 15 June 2024). We calculated the percentage of wound closure according to (Equation (1)) [51]:

(1) $$Wound\;Closure\;\left(\%\right)=\frac{A_{t=0}-A_{t=\Delta t}}{A_{t=0}}\times 100$$

where $A_{t=0}$ is the initial wound area, and $A_{t=\Delta t}$ is the wound area after n hours of the initial scratch.

The quantification of the wound area, wound coverage of the total area, average wound width, and width standard deviation in the images obtained from the wound-healing assay was carried out according to Suarez-Arnedo’s method [52]

**Figure 16:**
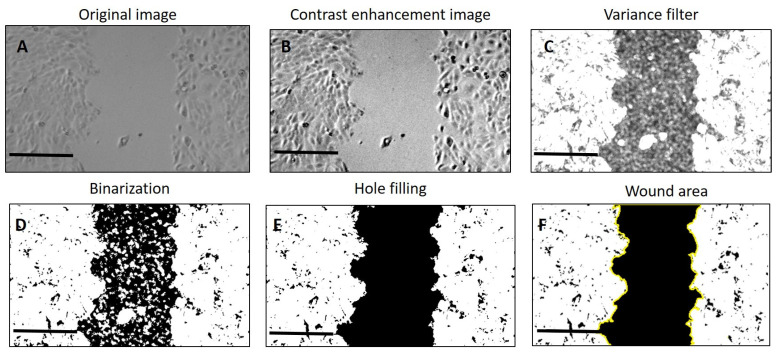
Overall process of wound image processing. (**A**) Original image; (**B**) scratch image after applying enhanced contrast; (**C**) image after applying the variance filter; (**D**) binarization of the image; (**E**) image after applying hole filling on the area of the wound; (**F**) selection of the scratch area.

The images were imported into the software, and a variance filter was applied (Figure 16C) after having enhanced contrast (Figure 16B) to create a new image with high-intensity pixels within the cell monolayer and low-intensity pixels within the open wound area. Afterward, we applied a threshold to the processed image in order to obtain binary segments (Figure 16D). Since the wound area can contain single cells or cell islets, we performed hole filling on the area of the wound (Figure 16E). Figure 16 presents an overview of the image-processing carried out to have a selection of the scratch area (Figure 16F).

The scratch area on the 0th hour was considered 100%, and the reduction in area was calculated for each set at 6, 16, and 24 h and plotted on a graph. The data were expressed as the mean ± SD. One-way ANOVA, followed by post hoc test, was used to compare the control (CTRL) and groups treated with PVA8/CMC3.5 and SER2 dressings. A *p*-value of ≤0.05 was considered statistically significant.

### 4.6. Ex Vivo Experiments

**Figure 17:**
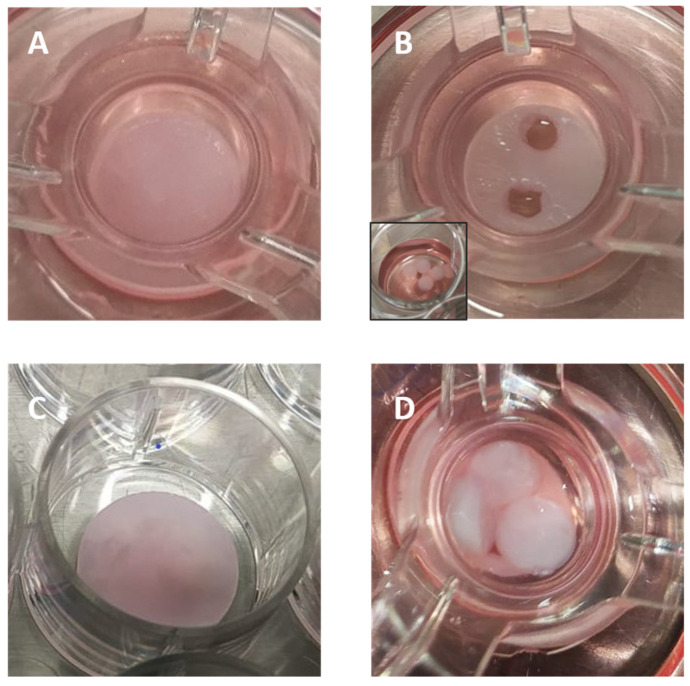
(**A**) EpiDermFT^TM^ tissue following wounding; (**B**) cut EpiDermFT^TM^ tissue; inset: sections of epidermis; (**C**) hydrogel wound healing dressing after immersion in DMEM medium; (**D**) cut EpiDermFT^TM^ tissue incubated with wound dressings.

Wound healing experiments were conducted in a full-thickness in vitro human skin model (EFT). This model exhibits stratified epidermal components and a fully developed basement membrane and resembles in vivo skin in regard to both morphology and barrier function. Furthermore, the cultures were derived from human neonatal foreskin tissue to form a multi-layered highly differentiated model of human skin that consisted of human-derived epidermal keratinocytes and human-derived dermal fibroblasts grown on a semi-permeable membrane. The EFT skin tissue inserts were transferred into 6-well plates containing 2.5 mL of serum-free medium upon arrival. The tissues were then equilibrated at 37 °C under 5% a CO_2_ atmosphere condition overnight. The medium was aspirated and replaced with fresh pre-warmed media after the equilibrations. The epidermal wounds were induced using a sterile 2 mm dermal biopsy punch (Figure 17B). After removing the cut sections of the epidermis with a fine tipped forceps, hydrogel wound healing dressings were added. The control experiments received only fresh pre-warmed serum-free medium.

Epithelial healing was observed within 5 days following the punch biopsy.

Following treatment, the EpiDermFT^TM^ tissues were fixed in 4% paraformaldehyde for 30 min and then washed three times with PBS containing 0.01% Triton X-100. The tissues were blocked for 1 h with 10% normal goat serum/1% BSA in PBS. The primary antibody was diluted in 1% BSA/PBS and incubated at room temperature for 2 h. Following incubation with the primary antibody, the tissues were washed two times (10 min for each wash) in PBS containing 0.1% normal goat serum. The secondary antibody (goat anti-mouse 488, AlexaFluor, Molecular Probes) was diluted 1:400 in 1% BSA/PBS and incubated with the samples for 1 h at room temperature. Following incubation with the secondary antibody, the tissues were rinsed with PBS containing 0.1% normal goat serum and stained with DAPI (0.1 μg/mL). The images were captured using Zeiss Axio Imager M2 (Carl Zeiss Microscopy, LLC, White Plains, NY, USA).

## Figures and Tables

**Table 1:** Different compositions of hydrogel wound healing dressings with different amounts (milligrams) of PVA, CMC, and sericin. The numbers appearing in the sample names for CMC and sericin (SER) correspond to the percentages with respect to the amount of PVA.

Sample Name	Composition (mG per G of Hydrogel)		
	PVA	CMC	Sericin
PVA10	100	0	0
PVA8	80	0	0
PVA6.7	67	0	0
PVA5	50	0	0
PVA8/CMC2	80	1.6	0
PVA8/CMC3.5	80	2.8	0
PVA8/CMC5	80	4	0
PVA8/CMC10	80	8	0
PVA8/SER2	80	0	1.6
PVA8/SER5	80	0	4
PVA8/SER10	80	0	8
PVA8/SER15	80	0	12
PVA8/SER25	80	0	20
PVA8/CMC3.5/SER2.5	80	2.8	2
PVA8/CMC3.5/SER5	80	2.8	4
PVA8/CMC3.5/SER7.5	80	2.8	6
PVA8/CMC3.5/SER10 (SER1)	80	2.8	8
PVA8/CMC3.5/SER15 (SER2)	80	2.8	12

## Data Availability

The data presented in this study are openly available in article.
